# Typhus and Other Pestilences

**Published:** 1916-10-14

**Authors:** 


### EPIDEMICS RESULTING FROM WARS.*

### Typhus and other Pestilences.

The events of the past two years have tended to throw into obscurity such work as has been carried out under the Carnegie Endowment for International Peace. The publication of a monograph on epidemics resulting from wars, designed to bring into light an aspect of international conflict which has never been adequately appreciated, is, however, to-day not inopportune. There is yet no certain ground for the argument that, in view of the progress of modern medical science, a study of war epidemics can have an interest purely historical. Dr. Friedrich Prinzing's work is concerned not so much with epidemics occurring in armies in the field as with certain of the less frequently studied war epidemics amongst the non-belligerent part of the population.

The present war has already brought about, in Serbia, one such outbreak of disease: the probabilities of further increased morbidity amongst the civil population of the belligerent countries are not to be prognosticated. We ourselves, though we may have as a nation yet further cause to be thankful for our insular isolation, shall do well to be not yet over-confident in the protection from disease afforded by it.

### Battle, Murder, and Sudden Death.

The causes which have made war, famine, and pestilence a trinity of evils have never been far to seek; the reasons which conjoin war and pestilence have become ever easier of scientific explanation. Although any infective disease may spread in consequence of war conditions and become epidemic, the author applies the name "war pestilence" only to those which have in the course of centuries usually followed at the heels of belligerent armies—typhus fever, bubonic plague, cholera, enteric fever, dysentery, and smallpox. He also includes scurvy, remarking that in its causation "there is probably a specific infective agent," although its etiology "has not yet been definitely determined."

It must be acknowledged that by far the greater portion of this book is devoted to the consideration of epidemics in Continental Europe. The bulk of its information is derived from German sources, but it contains too much of interest to be discarded on that account or because of its occasionally wearisome statistical tone. Apart from a few pages relating to earlier and to later war pestilences and to epidemics in besieged strongholds, the periods dealt with are those of the Thirty Years' War and the Franco-German War of 1870-71, with briefer references to other wars during the intervening period. Information concerning the conditions of non-belligerent populations during wars previous to the seventeenth century, and the incidence of disease amongst them, is scanty; and, even until a much later date, there is difficulty in identifying in modern medical terms the nature of epidemics from such confused references to their symptomatic characters as are available. Typhus fever stands out as the typical and ever-recurring war pestilence. Medical history of the period of the Thirty Years' War is singularly lacking, most of the author's account being based upon the work of Lammert; but it is clear that typhus was the prevailing and specific epidemic disease. A study of the awful state of affairs in Germany at that time as here decribed may possibly serve to sustain and comfort present-day Germans in their comparatively petty physical war troubles. The ravages of typhus "caused the inhabitants of many provinces to remember with hatred and loathing the departed soldiers, who were usually accused of having planted the seed of death." The depopulation of Germany during the Thirty Years' War chiefly resulted from epidemics of typhus and bubonic plague; in the regions where the war was carried on for several years the population decreased by far more than one-half. "War typhus" is again described at Verdun in 1792-95; Neumann, a Saxon, and others made (in 1812) some interesting observations as to its infectivity; typhus it was which followed Napoleon's army to Moscow and back again. In Vilna at this time, as elsewhere, is noted the spread of the disease amongst the Jews, who got possession of the clothes of the dead. Typhus fever reached its high-water mark of prevalence in Central Europe at the beginning of the nineteenth century.

### Smallpox in 1870.

The longest section of the book is that devoted to the epidemics of smallpox which followed upon the Franco-German War of 1870-71; space does not permit of detailed reference here to these or to the briefer accounts given of conditions following upon later wars. That their sanitary lessons have been learnt will be further shown by medical history now in the making.

This well-printed book, containing (as it does) a fairly full index, is a useful collection of object-lessons. Even though it be to-day rather historical than instructive, it is of great interest, and forms a valuable work of reference. As a painful arraignment of one aspect of the horrors and calamity of war in the past it is irrefutable and its aim is achieved.

* *Epidemics Resulting from Wars.* By Dr. Friedrich Prinzing. (Carnegie Endowment for International Peace.) Oxford: Clarendon Press. 1916. Pp. 340. 7s. 6d. net.

